# Supplementary material for: Epigenetic activation of the FLT3 gene by ZNF384 fusion confers a therapeutic susceptibility in acute lymphoblastic leukemia
Source: Nat Commun. 2022 Sep 14;13:5401. doi: 10.1038/s41467-022-33143-w (PMC9474531; doi:10.1038/s41467-022-33143-w)
Supplement: Supplementary file 1 — Supplementary Information file [file 41467_2022_33143_MOESM1_ESM.pdf]

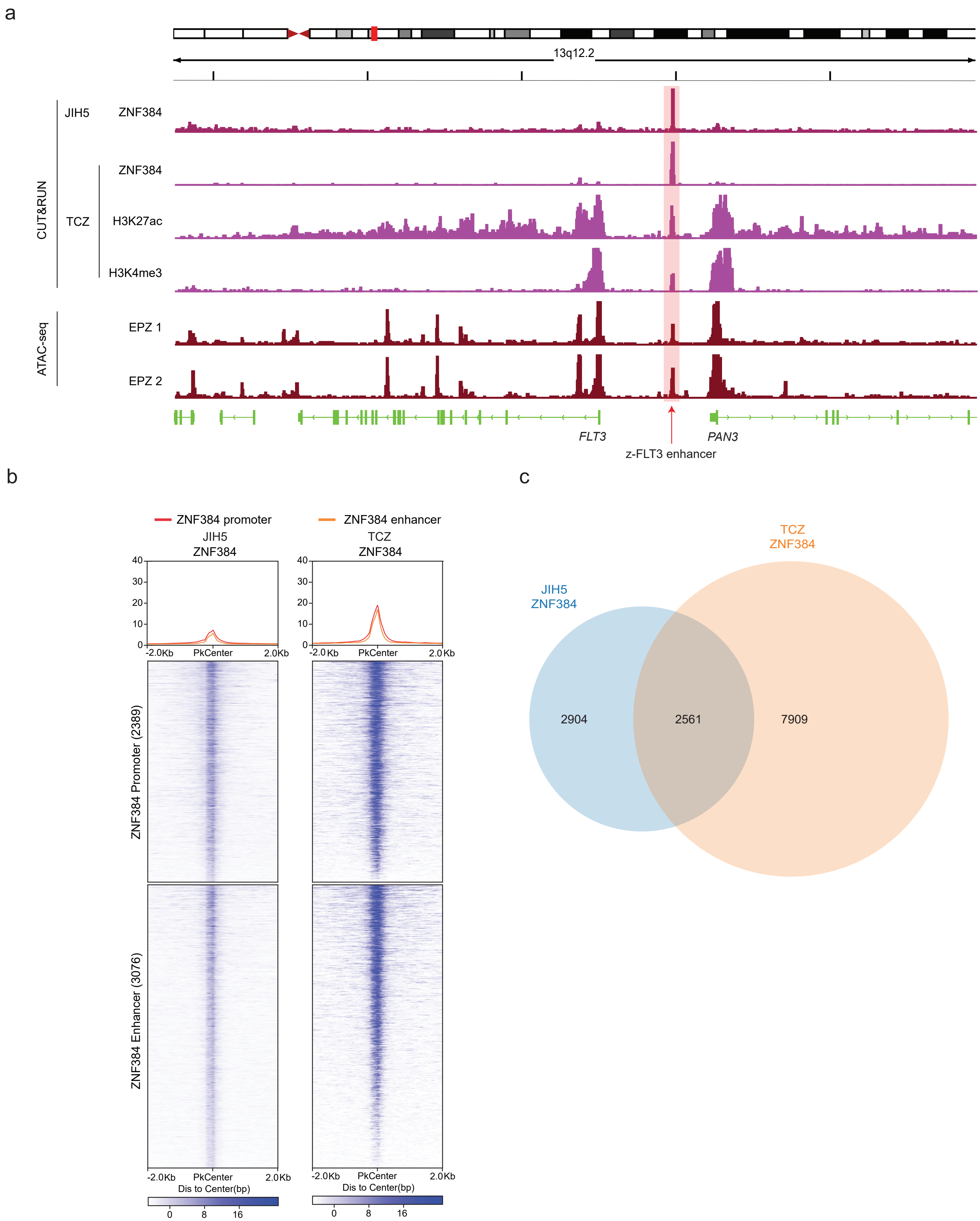

Supplementary Fig. 1: ZNF384 CUT&RUN in JIH5 cells. a ZNF384 binding profiling was characterized by ZNF384 CUT&RUN in JIH5 cells (top panel). A specific binding peak was observed at the upstream of *FLT3* gene, overlapping with the z-FLT3 enhancer identified in *TCF3-ZNF384* ALL PDX sample (TCZ). **b** In ZNF384 CUT&RUN assay using JIH5 cells, a total of 5,465 peaks were identified, with 2,389 in promoter regions (+/- 2kb from TSS) and the other 3,076 in enhancers. **c** Of the ZNF384 binding peaks in JIH5 cells, 2,561 peaks were overlapped with that in TCZ sample. Source data are provided as a Source Data file.

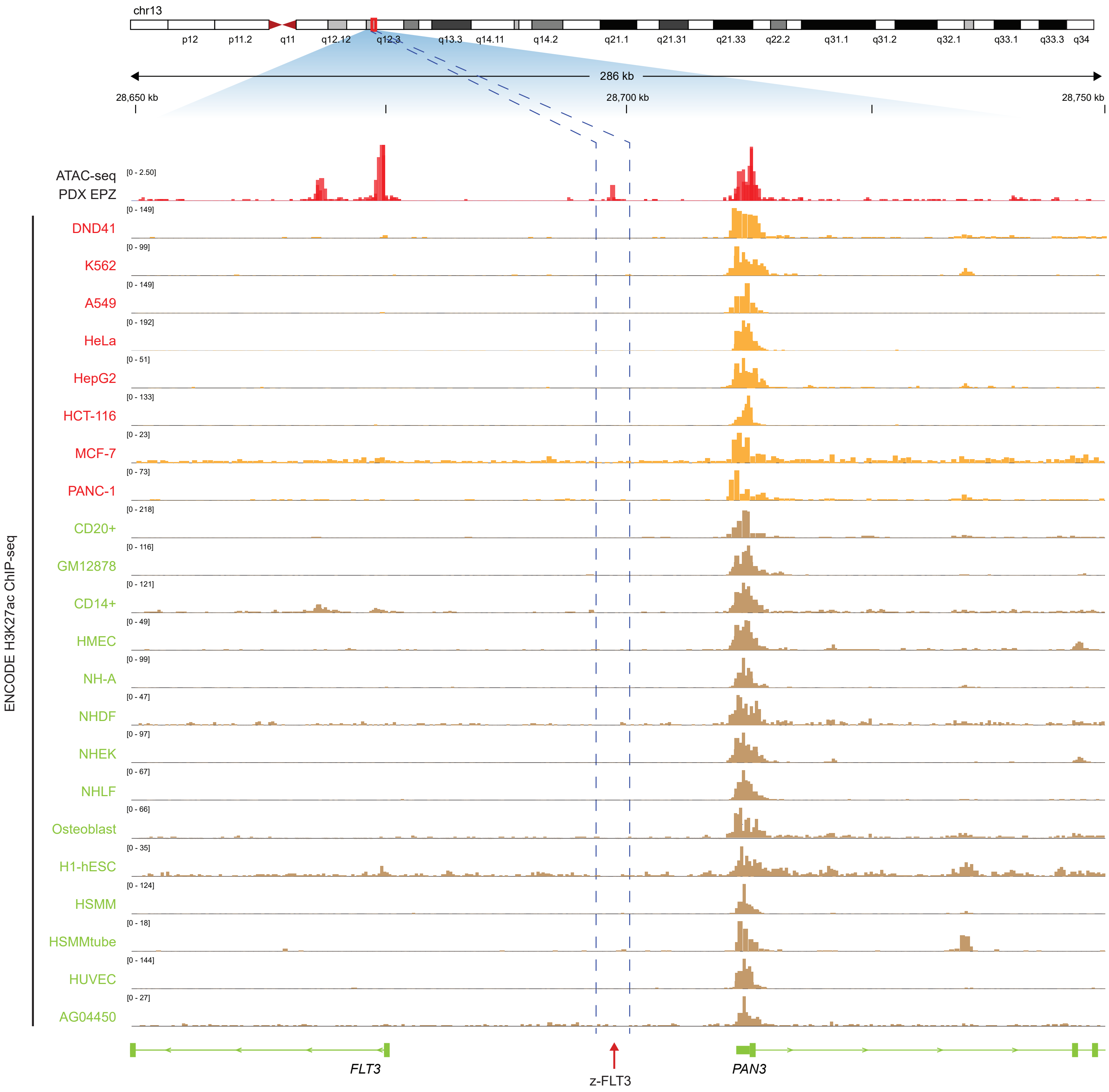

Supplementary Fig. 2: z-FLT3 enhancer is unique to ZNF384-r ALL. Exploring all the available H3K27ac ChIP-seq data of a series of 8 cancer cell lines and 14 normal human cells/tissues included in ENCODE, we did not observe the z-FLT3 enhancer (top track, ATAC-seq data from PDX EPZ) in any of them. Source data are provided as a Source Data file.

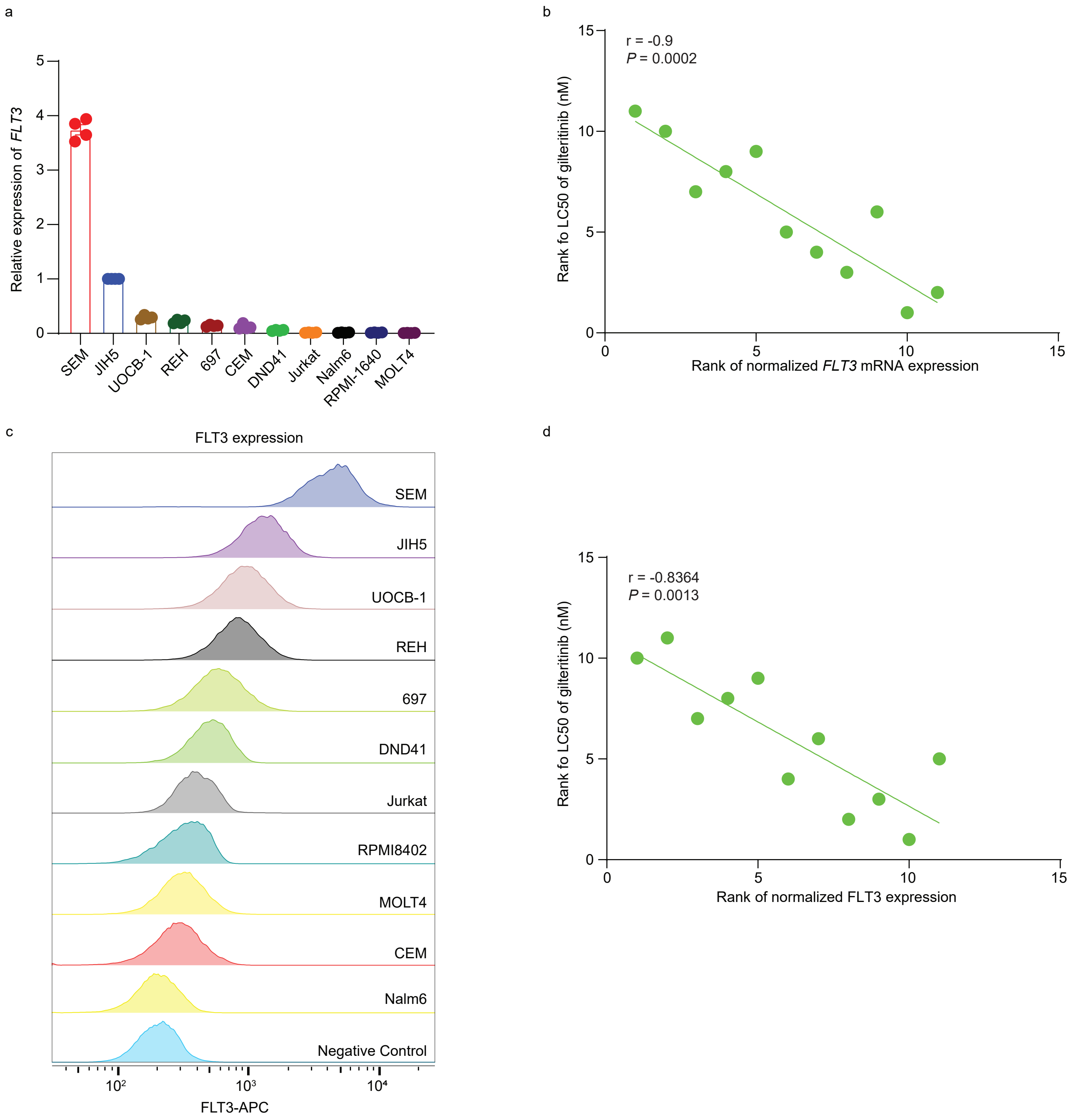

Supplementary Fig. 3: Cytotoxic effect of gilteritinib is correlated with FLT3 expression in ALL cell lines. a *FLT3* expression of 11 ALL cells was determined by RT-PCR. Data points represent relative expression of *FLT3* normalized to that of JIH5 cells. Data are shown as mean values ± SEM of four biological replicates (center of the error bar) and results are reprensentative of three independent experiments. **b** A negative correlation between LC50 of gilteritinib and *FLT3* expression was evaluated using two-sided Pearson correlation test (r = -0.9, *P* = 0.0002). **c** FLT3 expression on the surface of 11 ALL cells was determined by flow cytometry. **d** A negative correlation between LC50 of gilteritinib and FLT3 expression was assessed using two-sided Pearson correlation test (r = -0.8364, *P* = 0.0013). Source data are provided as a Source Data file.

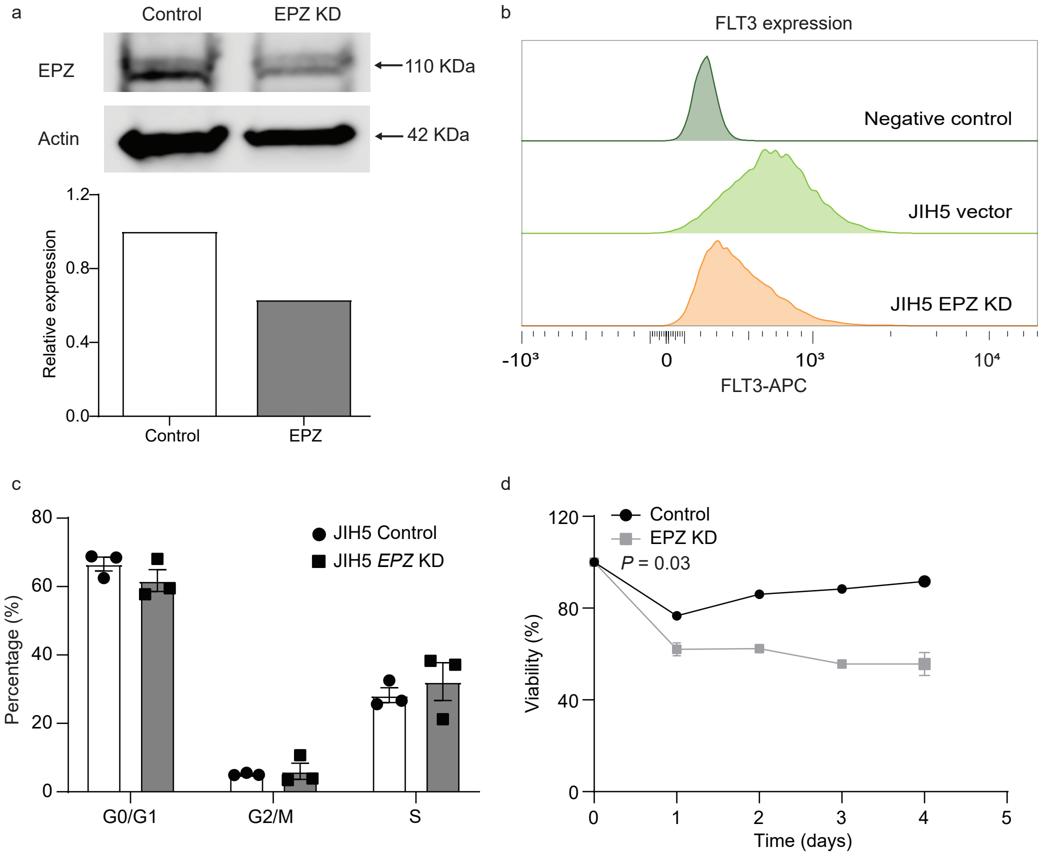

Supplementary Fig. 4: Knocking down *EPZ* fusion gene led to decreased FLT3 expression and cell viability in JIH5 cells. a Down regulation of EPZ fusion protein by Cas9/CRISPR was confirmed by Western blot, and quantified results were shown in the bar graph after normalizing to actin. Three independent experiments (biological replicates) were performed showing similar results. **b** Knocking down EPZ fusion protein led to down-regulated FLT3 expression on the surface of JIH5 cells, evaluated by flow cytometry. **c-d** Knocking down EPZ fusion protein didn't change the cell cycle distribution (**c**) but led to decreased cell viability of JIH5 cells (**d**). Data are shown as mean values ± SEM of three biological replicates (center of the error bar) and results are reprensentative of three independent experiments. *P* value ($P = 0.03$) was estimated using two-sided *t*-test. Source data are provided as a Source Data file.

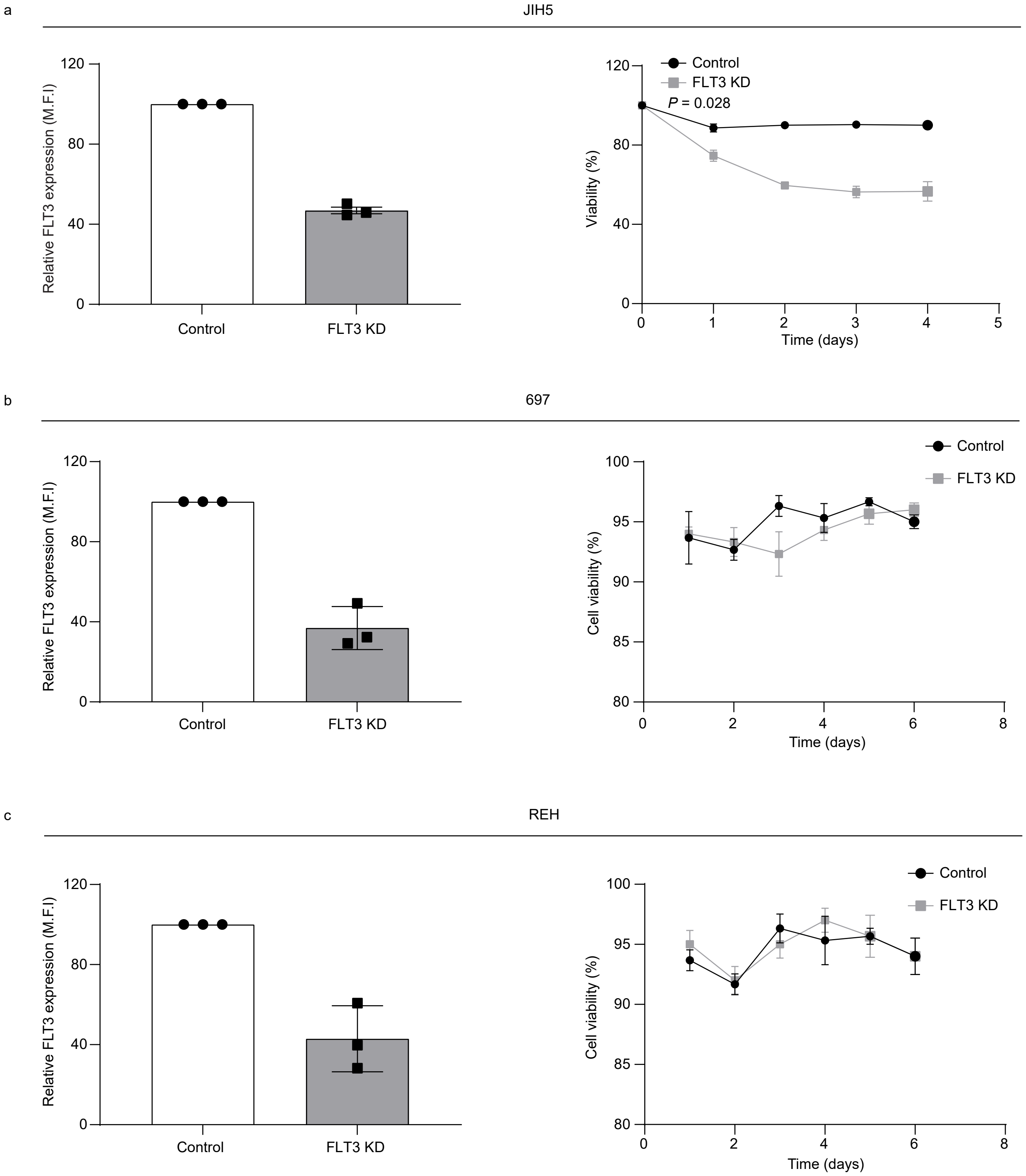

Supplementary Fig. 5: Knocking down FLT3 led to decreased cell viability in JIH5 cells. Down regulation of *FLT3* (using siRNA) led to decreased cell viability of JIH5 cells (**a**), but knocking down *FLT3* (using dCas9/CRISPR) didn't alter the viability of 697 (**b**) and REH cells (**c**). Data are shown as mean values ± SEM of three biological replicate (center of the error bar) and results are representative of three independent experiments. *P* value ($P = 0.028$) of panel **a** was estimated using two-sided *t*-test. M.F.I., Mean Fluorescent Intensity. Source data are provided as a Source Data file.

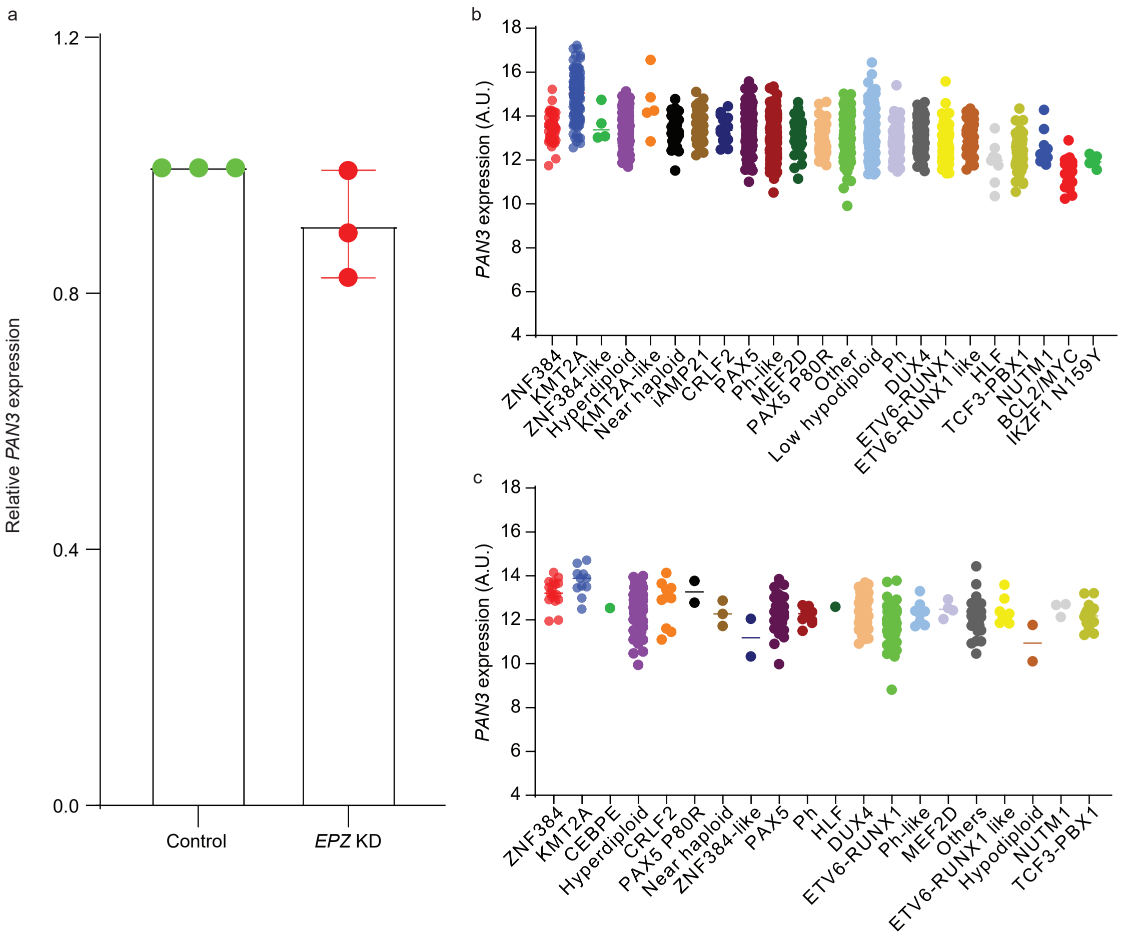

Supplementary Fig. 6: Expression of *PAN3* was not regulated by ZNF384 fusion gene. a Expression of *PAN3*, the nearby gene of *FLT3*, was not significantly altered after downregulation of *EP300-ZNF384* fusion gene in JIH5 cells. Expression was determined by qRT-PCR and normalized to control. Data are shown as mean values ± SEM of three biological replicates (center of the error bar) and results are representative of three independent experiments. **b** *PAN3* expression across different B-ALL subtypes in the US cohort of 1,988 children and adults (n = 1,988). **c** *PAN3* expression across different B-ALL subtypes in the Asian cohort (n = 377). A.U., arbitrary units. Center lines of panels **b** and **c** indicate median values of *PAN3* expression. Source data are provided as a Source Data file.

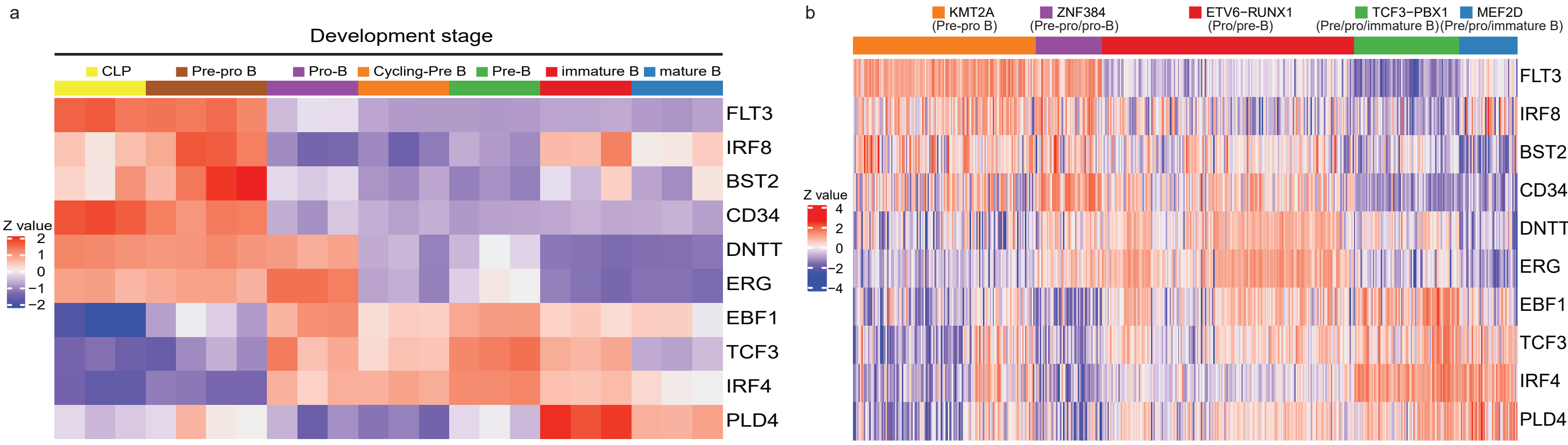

Supplementary Fig. 7: *FLT3* expression in ALL is linked to B-cell differentiation stages. a Seven stages of B-cell differentiation were identified using expression of 10 marker genes (*FLT3*, *IRF8*, *BST2*, *CD34*, *DNTT*, *ERG*, *EBF1*, *TCF3*, *IRF4*, and *PLD4*) (Michio W Painter, et al., J Immunol. 2011 Mar 1;186(5):3047-57.) on the basis of analyzing genome-wide gene expression data of normal hematopoietic cells (dataset: A comprehensive single cell transcriptional landscape of human hematopoietic cells [https://data.humancellatlas.org/explore/projects/5116c081-8be7-49c5-8ce0-73b887328aa9]). **b** Based on the expressions of these 10 genes, *KMT2A*-r subtype was defined as Pre-pro B, *ZNF384*-r subtype as Pre-pro or pro-B, *ETV6-RUNX1* subtype as Pro-or Pre-B, *TCF3-PBX1* and *MEF2D* subtypes as Pro- or pre- or immature B.

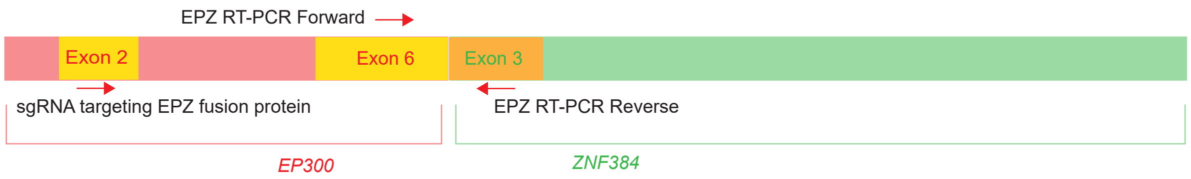

Supplementary Fig. 8: Positions of sgRNA and primers targeting *EPZ* fusion gene. The relative positions of sgRNA targeting *EPZ* fusion gene and primers for *EPZ* fusion gene RT-PCR were indicated by arrows.

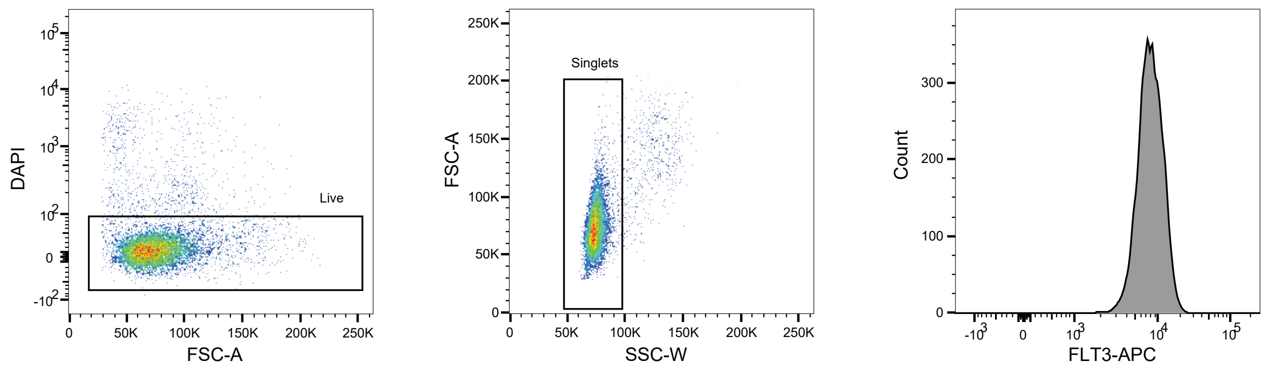

Supplementary Fig. 9: Gating strategy for determining expression of FLT3 on the cell surface of leukemia cells. Leukemia cells were stained with FLT3-APC antibody. DAPI and FSC-A were used to determine live cells. FSC-A and SSC-W were used to determine singlets. FLT3-APC positive cells were gated to determine the expression of FLT3 on the cell surface.

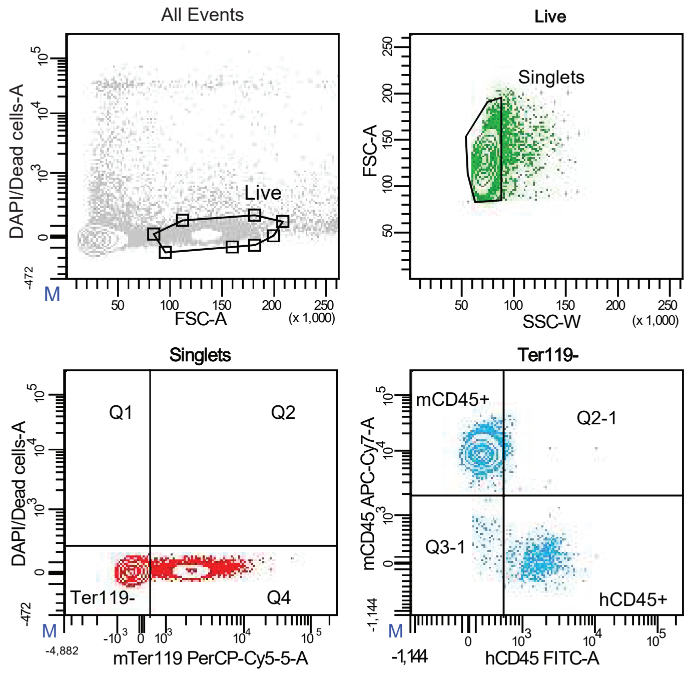

Supplementary Fig. 10: Gating strategy for determining human blast percentage in PDX mouse. Mouse retro-orbital blood was RBC lysed and then analyzed by flow cytometry. FSC-A/DAPI was used to determine live cells, and SSC-W/FSC-A was used to determine singlets. DAPI and TER119 were used to determine singlet live lymphocytes (non-red blood cells). Then hCD45 and mCD45 were used to determine human blast percentage.

Supplementary Table 1 : sgRNA for CRISPR/Cas9 knocking out *EP300-ZNF384* fusion gene

Name	Forward	Reverse
sgRNA	CTGTCAGAATTGCTGCGATC	GATCGCAGCAATTCTGACAG

Supplementary Table 2 : Qauntitative real-time PCR primers for *EP300-ZNF384* fusion gene, *FLT3* , and *PAN3*

Name	Forward	Reverse
FLT3	AGGGACAGTGTACGAAGCTG	GCTGTGCTTAAAGACCCAGAG
EP300-ZNF384	AGCTCTTGGACTACCCTATCA	TTCTCGATCTGACCTGAGACT
PAN3	CCGCTACTACGCTAAGGATAAGA	GGGACGCTGTTGCTATGGAG
S18	AGGAGCGATTTGCTGGTGTG	GCTACCAGGGCCTTTGAGAT

Supplementary Table 3. Information (sex and age) of participants in *ex vivo* drug sensitivity assay

Subtype		LC50 of gilteritinib (nM)	Gender	Age (year)
BCR-ABL1	Case 1	695.59	NA	>18
	Case 2	1032.653	NA	>18
DUX4	Case 1	128.852	Female	>18
	Case 2	199.61	Male	<10
	Case 3	233.906	Male	<10
	Case 4	245.244	Female	>10
	Case 5	824.083	Male	>10
	Case 6	1005.404	NA	>18
ETV6-RUNX1	Case 1	139.049	Female	<10
	Case 2	159.316	Female	<10
	Case 3	164.903	Male	<10
	Case 4	527.807	Male	<10
	Case 5	689.881	Male	<10
	Case 6	786.77	Female	<10
	Case 7	817.807	Male	<10
	Case 8	847.368	Female	>10
	Case 9	928.041	Male	<10
	Case 10	1214.616	NA	>18
	Case 11	1294.78	NA	>18
	Case 12	20000	NA	>18
Hyperdiploid	Case 1	147.942	Female	<10
	Case 2	342.092	Female	<10
	Case 3	853.5	Male	<10
	Case 4	862.059	Male	<10
	Case 5	955.324	Male	<10
	Case 6	1117.684	Female	<10
	Case 7	5578.25	NA	>18
	Case 8	20000	Female	<10
	Case 9	20000	NA	>18
T-ALL	Case 1	474.794	NA	>18
	Case 2	831.35	Female	<10
	Case 3	863.692	Male	<10
	Case 4	904.718	Female	<10
	Case 5	1047.975	NA	>18
	Case 6	2510.443	NA	>18
	Case 7	3962.696	NA	>18
	Case 8	20000	NA	>18
	Case 9	20000	NA	>18
TCF3-PBX1	Case 1	486.559	Male	<10
	Case 2	634.937	Female	<10
	Case 3	952.715	Male	<10
	Case 4	983.779	Male	>10
	Case 5	1001.538	Female	>10
	Case 6	1038.442	Male	>10
ZNF384	Case 1	4.885	Male	<10
	Case 2	4.885	Male	>18
	Case 3	17.948	Female	>18
