# Supplementary material for: Epigenetic activation of the FLT3 gene by ZNF384 fusion confers a therapeutic susceptibility in acute lymphoblastic leukemia
Source: Nat Commun. 2022 Sep 14;13:5401. doi: 10.1038/s41467-022-33143-w (PMC9474531; doi:10.1038/s41467-022-33143-w)
Supplement: Supplementary file 3 — Reporting Summary [file 41467_2022_33143_MOESM3_ESM.pdf]

nature portfolio

Corresponding author(s): Jun J. Yang

Last updated by author(s): Aug 30, 2022

Reporting Summary

Nature Portfolio wishes to improve the reproducibility of the work that we publish. This form provides structure for consistency and transparency in reporting. For further information on Nature Portfolio policies, see our Editorial Policies and the Editorial Policy Checklist.

Statistics

For all statistical analyses, confirm that the following items are present in the figure legend, table legend, main text, or Methods section.

n/a	Confirmed	
☐	☒	The exact sample size (n) for each experimental group/condition, given as a discrete number and unit of measurement
☐	☒	A statement on whether measurements were taken from distinct samples or whether the same sample was measured repeatedly
☐	☒	The statistical test(s) used AND whether they are one- or two-sided<br>*Only common tests should be described solely by name; describe more complex techniques in the Methods section.*
☐	☒	A description of all covariates tested
☒	☐	A description of any assumptions or corrections, such as tests of normality and adjustment for multiple comparisons
☐	☒	A full description of the statistical parameters including central tendency (e.g. means) or other basic estimates (e.g. regression coefficient) AND variation (e.g. standard deviation) or associated estimates of uncertainty (e.g. confidence intervals)
☐	☒	For null hypothesis testing, the test statistic (e.g. F, t, r) with confidence intervals, effect sizes, degrees of freedom and P value noted<br>*Give P values as exact values whenever suitable.*
☒	☐	For Bayesian analysis, information on the choice of priors and Markov chain Monte Carlo settings
☒	☐	For hierarchical and complex designs, identification of the appropriate level for tests and full reporting of outcomes
☐	☒	Estimates of effect sizes (e.g. Cohen's d, Pearson's r), indicating how they were calculated

Our web collection on statistics for biologists contains articles on many of the points above.

Software and code

Policy information about availability of computer code

Data collection	See below for description of collection (and analyses, full description of data collection and analyses can be found in methods).<br>Flow cytometry (FACS): BD FACS Diva Software V8.0; FlowJo software V10.5.3/10.6.1;<br>Ex vivo leukemia drug-sensitivity assay: PerkinElmer Harmony high-content imaging and analysis software (version 4.9).<br>Sequencing (CUT&RUN, ATAC-seq, and ChIA-PET): HiSeq 2000/2500 or NovaSeq 6000 platform;<br>MTT (measurement of MTT absorbance) : BioTek Gen5 software
Data analysis	ATAC-seq data was analyzed as below: Paired-end reads were applied on cutadapt (version 1.18) for adaptor trimming, and then mapped to the human hg19 genome reference by Bowtie2 (version 2.2.9). Peak calling was performed by MACS230 with default parameters on each sample. Peaks from all samples were merged by bedtools (version 2.25.0) to retain non-overlapped regions. These regions were then used for identifying differentially enriched peaks, which was performed by ABSSeq under the aFold model with read count from HTSeq. The cutoff of adjusted P values <0.05 and log2 fold change >=2 was used to define high confidence ATAC-seq peaks. Enriched regions were mapped to the nearest gene in human hg19 by Homer.<br>CUT&RUN data was analyzed as below: Paired-end reads of 100bp were mapped human genome hg19(GRCh37-lite)by BWA (version 0.7.12-r1039, default parameter) after trimming for adapters by fastp(version 0.20.0, paired-end mode, parameters "--trim_poly_x --cut_by_quality5 --cut_by_quality3 --cut_mean_quality 15 --length_required 20 --low_complexity_filter --complexity_threshold 30 --detect_adapter_for_pe"), duplicated reads were then marked with biobambam2(version v2.0.87) and only non-duplicated reads have been kept by samtools (parameter "-q 1 -F 1804" version 1.2). All samples have more than 5M fragments as suggested by Cut&Run protocols paper. We then generated bigwig files using the center 80bp of fragments smaller than 2000bp and normalized to 10M fragments. Peaks were called by MACS2 (version 2.1.1.20160309 , "--extsize 200 --nomodel --keep-dup all").<br>ChIA-PET data was processed and analyzed by ChIA-PIPE (Version 1.0). Code of ChIA-PIPE can be found at https://github.com/TheJacksonLaboratory/ChIA-PIPE.<br>MTT data was analyzed by Graphpad Prism (version 9).<br>Cell viability data for ex vivo leukemia drug-sensitivity assay were analyzed by Harmony high-content imaging and analysis software (version

4.9).

For manuscripts utilizing custom algorithms or software that are central to the research but not yet described in published literature, software must be made available to editors and reviewers. We strongly encourage code deposition in a community repository (e.g. GitHub). See the Nature Portfolio guidelines for submitting code & software for further information.

Data

Policy information about availability of data

All manuscripts must include a data availability statement. This statement should provide the following information, where applicable:

- Accession codes, unique identifiers, or web links for publicly available datasets
- A description of any restrictions on data availability
- For clinical datasets or third party data, please ensure that the statement adheres to our policy

All the sequencing data (CUT&RUN, ATAC-seq, and ChIA-PET) were uploaded to GEO (GSE197890 [https://www.ncbi.nlm.nih.gov/geo/query/acc.cgi?acc=GSE197890]). Previously published RNA-seq data can be available at EGAD00001004461 (https://ega-archive.org/datasets/EGAD00001004461), EGAD00001004463 (https://ega-archive.org/datasets/EGAD00001004463), EGAS00001001858 (https://ega-archive.org/studies/EGAS00001001858), EGAS00001003726 (https://ega-archive.org/studies/EGAS00001003726) and EGAS00001004532 (https://ega-archive.org/studies/EGAS00001004532). The data generated in this work are provided in the Supplementary Information and Source Data files.

Field-specific reporting

Please select the one below that is the best fit for your research. If you are not sure, read the appropriate sections before making your selection.

☒ Life sciences ☐ Behavioural & social sciences ☐ Ecological, evolutionary & environmental sciences

For a reference copy of the document with all sections, see nature.com/documents/nr-reporting-summary-flat.pdf

Life sciences study design

All studies must disclose on these points even when the disclosure is negative.

Sample size	Sample size was determined on the basis of data and sample availability
Data exclusions	No data was excluded
Replication	All in vitro experiments have been individually repeated at least three times. All attempts of replication were successful. In vivo experiments were repeated twice with similar results.
Randomization	To test the cytotoxic effect of treating ZNF384-r ALL with gilteritinib in vivo, NSG mice were randomly assigned to treatment or vehicle group. Only females mice with an age of 8 weeks were used to ensure maximal engraftment. Cages were placed on the same rack in the same room and were supplemented with equal food and water. Randomization is not applicable for in vitro experiment.
Blinding	In the in vivo gilteritinib efficacy testing, human blast percentage of all samples were determined before we matched the number to each particular mouse. We wouldn't know the identification of each mouse until the human blast percentage has been matched to each individual mouse. Blinding is not applicable for in vitro experiment.

Reporting for specific materials, systems and methods

We require information from authors about some types of materials, experimental systems and methods used in many studies. Here, indicate whether each material, system or method listed is relevant to your study. If you are not sure if a list item applies to your research, read the appropriate section before selecting a response.

Materials & experimental systems

n/a	Involved in the study	
☐	☒	Antibodies
☐	☒	Eukaryotic cell lines
☒	☐	Palaeontology and archaeology
☐	☒	Animals and other organisms
☐	☒	Human research participants
☒	☐	Clinical data
☒	☐	Dual use research of concern

Methods

n/a	Involved in the study	
☒	☐	ChIP-seq
☐	☒	Flow cytometry
☒	☐	MRI-based neuroimaging

Antibodies

Antibodies used	ZNF384 (Abclonal, #A15964, dilution ratio: 1:500 for Western blot, four ug for CUT&RUN), H3K27ac (CST, #817S, four ug), H3K4me3

	(CST, #9751, four ug), CTCF (Abclonal, #1133, 20 ug), RNAPII (BioLegend, #664906, 20 ug), Actin (CST, #4967, dilution ratio: 1:2,000), FLT3 (Invitrogen, #17-1357-42, dilution ratio: 1:100), mTER119-PerCP-Cy5.5 (BD Pharmingen, #560512, Clone TER-119, dilution ratio: 1:100), mCD45-APC-Cy7 (BD Pharmingen, #557659, Clone 30-F11, dilution ratio: 1:100), CD45-FITC (BD Pharmingen, #555482, Clone HI30, dilution ratio: 1:100)
Validation	ZNF384 (Abclonal #A15964) was validated in human cells by Western blot<br>https://abclonal.com/catalog-antibodies/ZNF384RabbitpAb/A15964<br>H3K27ac (CST#817S) was validated in human cells by Western blot<br>https://www.cellsignal.com/products/primary-antibodies/acetyl-histone-h3-lys27-d5e4-xp-rabbit-mab/8173<br>H3K4me3 (CST#9751) was validated in human cells by Western blot<br>https://www.cellsignal.com/products/primary-antibodies/tri-methyl-histone-h3-lys4-c42d8-rabbit-mab/9751<br>CTCF (Abclonal #1133) was validated in human cells by Western blot<br>https://abclonal.com/catalog-antibodies/CTCFRabbitpAb/A1133<br>RNAPII (BioLegend #664906) was validated in human cells by Western blot<br>https://www.biolegend.com/en-us/products/purified-anti-rna-polymerase-ii-rpb1-antibody-11666<br>Actin (CST #4967) was validated in human cells by Western blot<br>https://www.cellsignal.com/products/primary-antibodies/b-actin-antibody/4967<br>FLT3 (Invitrogen #17-1357-42) was validated in human cells by Flow cytometry<br>https://www.thermofisher.com/antibody/product/CD135-Flt3-Antibody-clone-BV10A4H2-Monoclonal/17-1357-42<br>CD45-FITC (BD Pharmingen, #555482, Clone HI30) was validated in human cells by Flow cytometry<br>https://www.bdbiosciences.com/en-au/products/reagents/flow-cytometry-reagents/research-reagents/single-color-antibodies-ruo/fitc-mouse-anti-human-cd45.555482<br>mouse ER119-PerCP-Cy5.5 (BD Pharmingen, #560512, Clone TER-119) was validated in mouse cells by Flow cytometry<br>https://www.bdbiosciences.com/en-ca/products/reagents/flow-cytometry-reagents/research-reagents/single-color-antibodies-ruo/percp-cy-5-5-rat-anti-mouse-ter-119-erythroid-cells.560512<br>mCD45-APC-Cy7 (BD Pharmingen, #557659, Clone 30-F11) was validated in mouse cells by Flow cytometry<br>https://www.bdbiosciences.com/en-us/products/reagents/flow-cytometry-reagents/research-reagents/single-color-antibodies-ruo/apc-cy-7-rat-anti-mouse-cd45.557659

Eukaryotic cell lines

Policy information about cell lines

Cell line source(s)	Nalm6, REH, RS 4:11, MOLT4, CEM, and Jurkat cell lines were purchased from ATCC, and JIH5, SEM, 697, RPMI8402, and DND41 cell lines were purchased from DSMZ. UOCB-1 is a kind gift from Dr. William Evans at St. Jude Children's Research Hospital, detailed information of this cell line is available at Cellosaurus database with accession number: CVCL_A296
Authentication	We have done authentication for all the cell lines used in this study by STR analysis
Mycoplasma contamination	All cell lines are negative for Mycoplasma
Commonly misidentified lines (See ICLAC register)	No commonly misidentified lines were used in this study

Animals and other organisms

Policy information about studies involving animals; ARRIVE guidelines recommended for reporting animal research

Laboratory animals	This study involved NOD.Cg-Prkdcscid Il2rgtm1Wjl/SzJ (NSG) mice, all were female and 8-12 weeks old
Wild animals	This study didn't involve any wild animals.
Field-collected samples	This study didn't involve any field-collected samples.
Ethics oversight	The study was conducted under the ethical approval for animal research by the Animal Care & Use Committee (ACUC) of St. Jude Children's Research Hospital.

Note that full information on the approval of the study protocol must also be provided in the manuscript.

Human research participants

Policy information about studies involving human research participants

Population characteristics	For Patients involved in the ex vivo drug sensitivity study include both children and adults, male and female, consistent with the age and gender distribution of ALL. Detail information (sex and age) of each participant are included in the Methods and Supplementary Table 3
Recruitment	Patients with ALL were included in this study primarily through the participation of the therapeutic trials and/or tissue banking protocols. Patients were not specifically recruited for the purpose of this research. Individuals were included he ex vivo drug sensitivity study solely based on sample availability. We are not aware of any bias in recruitment and sampling, and we do not believe our results are impacted by any sampling bias.

Ethics oversight | This study was reviewed and approved by institutional review boards at St. Jude Children's Research Hospital.

Note that full information on the approval of the study protocol must also be provided in the manuscript.

Flow Cytometry

Plots

Confirm that:

- [x] The axis labels state the marker and fluorochrome used (e.g. CD4-FITC).
- [x] The axis scales are clearly visible. Include numbers along axes only for bottom left plot of group (a 'group' is an analysis of identical markers).
- [x] All plots are contour plots with outliers or pseudocolor plots.
- [x] A numerical value for number of cells or percentage (with statistics) is provided.

Methodology

Sample preparation	The description of sample preparation can be found in the supplementary methods section. For flow analyses of cell lines and PDX cells from PDX mouse model, cells were resuspended in FACS buffer (PBS supplemented with 2% Fetal Calf Serum and 0.25mM EDTA), they were then stained with proper antibodies and washed twice with FACS buffer before being subjected to flow analyses.
Instrument	BD FACS Aria IIIu machine,
Software	BD FACS Diva Software and FlowJo.
Cell population abundance	Only populations with sufficient number of cells (event) were analyzed post-sorting.
Gating strategy	To determine human blast% in PDX samples, 30-50 uL mouse peripheral blood was collected. after RBC lysis, FSC-A/SSC-A was used to determine lymphocytes, after gating for live lymphocytes (DAPI/mTER119 double negative), SSC-W/FSC-A was used to determine singlets, then hCD45/mCD45 was used to determine human blast%, mCD45 was used to confirm.

- [x] Tick this box to confirm that a figure exemplifying the gating strategy is provided in the Supplementary Information.
